# Disruption of dnaK results in transcriptional reprogramming of biofilm-associated pathways in Clostridioides difficile

**DOI:** 10.1099/jmm.0.002212

**Published:** 2026-10-07

**Authors:** Megan G. Taggart, Carla S. Möller-Levet, William J. Snelling, Patrick J. Naughton, Roberto M. La Ragione, Barry M. G. O'Hagan, James S. G. Dooley, Nigel G. Ternan

**Affiliations:** 1 Nutrition Innovation Centre for Food & Health (NICHE), Ulster University, Coleraine, BT52 1SA, UK; 2 Faculty of Health and Medical Sciences, University of Surrey, Guildford, Surrey GU2 7XH, UK; 3 School of Biosciences, University of Surrey, Guildford, Surrey GU2 7XH, UK; 4 School of Veterinary Medicine, University of Surrey, Guildford, Surrey GU2 7AL, UK; 5 Genomic Medicine Research Group, Ulster University, Coleraine, BT52 1SA, UK

**Keywords:** biofilm, *Clostridioides difficile*, *dnaK*, stress response

## Abstract

**Introduction.** *Clostridioides difficile* is an opportunistic enteric pathogen posing significant challenges in human and animal healthcare due to the recurrent nature of infections and associated financial burden. The molecular chaperone protein, DnaK (Hsp70), encoded by *dnaK*, plays a central role in the heat stress response and protein homeostasis. Disruption of *dnaK* in *C. difficile* 630Δ*erm* has previously been associated with increased biofilm formation and multiple phenotypic alterations.

**Gap Statement.** Although disruption of *dnaK* has been shown to alter biofilm formation and multiple phenotypes in *C. difficile*, the global transcriptional mechanisms linking DnaK to biofilm development and stress adaptation remain poorly understood.

**Aim.** This study aimed to characterize the global transcriptional response associated with *dnaK* disruption during planktonic and biofilm growth to identify pathways linking the DnaK-mediated stress response with biofilm formation.

**Methods.** This study investigated the global transcriptional changes associated with *dnaK* disruption during planktonic and colony biofilm culture. Colony biofilms of *C. difficile* strains 630, 630Δ*erm* and 630Δ*erm:dnaK* were cultured on mixed nitrocellulose ester semi-permeable membranes and examined structurally using low-vacuum scanning electron microscopy. Total RNA was extracted from both planktonic cultures and 24 h colony biofilms, followed by Illumina RNA sequencing. Differential gene expression analysis was performed using DESeq (*P*_adj_<0.05). Gene set enrichment analysis (Kyoto Encyclopedia of Genes and Genomes [KEGG] and Gene Ontology) was conducted to identify significantly enriched metabolic pathways and biological processes.

**Results.** Disruption of *dnaK* resulted in global transcriptomic remodelling, particularly during biofilm culture, where 72.4% of genes were significantly differentially expressed relative to planktonic culture. A core number of pathways were involved in biofilm formation in all strains, including increased sporulation with reduced translation, amino acid biosynthesis and flagellar assembly. The *dnaK* mutant exhibited a unique pattern of enrichment of pathways associated with cell division, peptidoglycan synthesis, carbon metabolism and amino acid biosynthesis, alongside a coordinated reduction of motility and chemotaxis pathways.

**Conclusion.** Disruption of the *dnaK* gene appears to link the stress response to biofilm formation through a number of pathways associated with biofilm development. Understanding this link may identify systems relevant to persistent and recurrent infections.

## Data Summary

RNA sequencing data generated in this work have been deposited in the National Center for Biotechnology Information (NCBI) Gene Expression Omnibus (GEO) repository, accession number GSE336654. The authors confirm all supporting data, code and protocols have been provided within the article or through supplementary data files.

## Introduction

The opportunistic enteric pathogen *Clostridioides difficile* causes significant healthcare problems due to the recurrent nature of infections, becoming increasingly difficult to treat with each recurrence [1, 2]. The limited number of approved treatments for *C. difficile* infection (CDI) are threatened by the rise in antimicrobial resistance [3]. New treatment options are emerging, such as spore therapy, bezlotoxumab monoclonal antibody therapy and a glycopeptide antibiotic EVG7, which is a semisynthetic vancomycin derivative [4–6]. However, no single treatment has been successful in inhibiting disease recurrence in all cases; therefore, further understanding of the mechanism of recurrence is still necessary. Recurrence of infection has been attributed to the ability of *C. difficile* to form biofilms and spores within the gut [1, 2, 7]. Spores are important for the zoonotic and faecal–oral transmission and survival of *C. difficile* when exposed to oxygen, heat or disinfecting agents, allowing them to persist on hospital equipment [8–11]. Sporulation has been strongly associated with biofilm formation in *C. difficile* through the master regulator *Spo0A* [8]. However, the regulatory mechanisms of both processes are multifactorial and, particularly with respect to biofilm formation, not yet fully characterized. Multiple processes have been associated with biofilm formation in *C. difficile* in a complex web of interrelated pathways, which differ between strains [1, 2, 12]. The main systems that have been linked to biofilm formation in *C. difficile* include motility, sporulation and signalling by a second messenger molecule, c-di-GMP [1, 2, 12].

Initiation of biofilm formation in *C. difficile* has also been linked to stressors such as the presence of antimicrobials, including antibiotics such as vancomycin and metronidazole as well as heat stress [13]. A physiologically relevant increase in temperature from 37 to 41 °C, as would be notable in CDI, caused increased expression of Class I heat shock genes in *C. difficile* 630, which are involved in the stress response in *C. difficile* [14, 15]. Included in this response was increased expression of the *dnaK* gene (*CD2461*), found on the *dnaJK* operon, encoding the highly conserved DnaK molecular chaperone [14–16]. Also referred to as Hsp70 (heat shock protein 70), DnaK is responsible for protein folding, particularly under environmental conditions that cause stress to the cells. Disruption of the *dnaK* gene in *C. difficile* 630Δ*erm* using ClosTron mutagenesis led to various phenotypic changes in the mutant 630Δ*erm:dnaK* cells, including increased cell length by ~50%, loss of motility and increased aggregate biofilm production [17]. This identified the *dnaK* gene as a potential link between the bacterial stress response under physiologically relevant heat stress and biofilm formation, which is associated with increased disease severity. This work investigates transcriptomic changes in *dnaK*-deficient *C. difficile* in both planktonic and colony biofilms to identify pathways that may bridge the gap between heat stress and increased biofilm formation in *C. difficile*. We hypothesized that disruption of the *dnaK* gene would result in coordinated transcriptional changes involving pathways previously associated with biofilm formation, including sporulation, motility, cell wall remodelling and the stress response.

## Methods

### *C. difficile* strains and culture conditions

*C. difficile* strains 630 (ATTC: BAA-1382), 630Δ*erm* (originally gifted from Professor Minton’s Lab, University of Nottingham) and 630Δ*erm:dnaK* [17] were obtained from Ulster University frozen stocks maintained at −80 °C on TSC ‘Protect’ Anaerobe Cryobeads (Gem Scientific, UK). All strains were cultured on brain heart infusion (BHI) (37 g l^−1^) (OXOID, UK) agar (1.2% wt/v) supplemented with 5 g l^−1^ of yeast extract and 1 g l^−1^ of l-cysteine (BHIS) under anaerobic conditions (80% nitrogen, 10% hydrogen and 10% carbon dioxide) at 37 °C in a Don Whitley Scientific MACS500 anaerobic workstation. Overnight cultures were set up in triplicate for all experiments from a single colony in 10 ml of BHIS broth, preconditioned to anaerobic conditions and cultured for 16–18 h under anaerobic conditions.

### Colony biofilm growth on semi-permeable membranes

Colony biofilms of all strains 630, 630Δ*erm* and 630Δ*erm:dnaK* were cultured on white mixed nitrocellulose ester membranes (MF-Millipore, Germany) of pore size 0.22 µm (MF-Millipore, Germany) in a method adapted from James *et al*. (2018) [18]. The membranes were placed on BHIS agar, and a 30 µl volume of a 1% overnight bacterial inoculum in BHIS broth was carefully spotted to the centre of each membrane. All membrane biofilms were cultured for 24 h at 37 °C under anaerobic conditions. All assays were set up with three biological replicates, each with two technical replicates, for each strain, with three replicates of blank controls that were incubated only with sterile BHIS broth spotted onto the membranes.

### Low vacuum scanning electron microscopy of colony biofilms

Colony biofilms were removed from the anaerobic cabinet and fixed in 1 ml 2.5% glutaraldehyde in PBS (pH 7.4) for 1 h at room temperature. The biofilms were then rinsed by carefully submerging each in a separate 1 ml aliquot of 1× PBS (pH 7.4) in a 24-well microtitre plate. The membranes were returned to the original BHIS agar plate to reduce over-drying, while wet samples were being imaged. Samples were imaged under low vacuum using an FEI QuantaTM 200 (FEI Company, Eindhoven, The Netherlands) environmental scanning electron microscope (ESEM) equipped with a large field detector (LFD). All samples were processed using an accelerating voltage of 30 kV at spot size 4.0 and a working distance of 7.6–7.8 mm and a scan speed of 30 µs. Images were captured at 200×, 800× and 12,000× magnifications.

### RNA extraction

*C. difficile* colony biofilms grown on mixed nitrocellulose ester membranes (13 mm ø) for 24 h were used for total RNA extraction by the RNeasy PowerBiofilm kit (Qiagen, Germany). For each extraction, 6 biofilms were harvested by physically scraping them off the membrane using sterile plastic loops and into a solution of 350 µl MBL (RNeasy PowerBiofilm reagent) and 5 µl ß-mercaptoethanol (Sigma-Aldrich, US). The biofilm was homogenized in the solution by repeated pipetting up and down before being transferred into the bead tube. For planktonic cell harvest, a 10% inoculum from an overnight culture in 20 ml BHIS was cultured anaerobically for 6 h at 37 °C before 5.5 ml was centrifuged at 14,000 ×***g*** for 5 min. The supernatant was removed, and the cell pellets resuspended as per the biofilm samples in 350 µl solution containing 345 µl of solution MBL and 5 µl of ß-mercaptoethanol before being transferred to the bead tube. A 100 µl aliquot of FB solution from the RNeasy PowerBiofilm kit was also added to each bead tube before briefly vortexing and incubating at 65 °C for 5 min. Cell numbers were not quantified prior to RNA extraction due to the extraction kit specifying an input of ~50 mg biomass. Preliminary sacrificial biofilms and cultures were used to determine reproducible harvesting conditions that consistently yielded the required biomass (data not shown). The tubes were then vortexed horizontally using a vortex genie (Scientific Industries Inc., USA) with a horizontal adapter on full speed for 10 min. The bead tubes were then centrifuged for 1 min at 13,000 ***g*** and the supernatant transferred into a clean microtube. To the supernatant, 100 µl of ice-cold IRS solution was added and mixed by inverting several times before being placed on ice for 5 min at 4 °C. The microtubes were centrifuged as before for 1 min at 13,000 ×***g*** at room temperature, and the supernatant was transferred into a new microtube. To the supernatant, 450 µl of PB solution was added as well as 450 µl of ethanol. The solution was mixed by pipetting up and down until uniformly mixed before being transferred into spin columns in 650 µl portions and centrifuged as before until all the solution had been used and the flow-through discarded. The spin columns were washed with 650 µl of well-shaken PW solution centrifuged through the column at 13,000 ×***g*** for 1 min, and the flow-through discarded. The columns were then centrifuged again as before with no additional reagent before 50 µl of DNase and RDD buffer supplied in the kit were added and incubated for 20 min at room temperature. To the columns containing DNase solution, 400 µl of WB buffer was added and the columns centrifuged as before and the flow-through discarded. The columns were then washed once more as before with 650 µl of PW solution. Aliquots of 650 µl of ethanol were then added to the column, centrifuged as before, and the flow-through discarded. To remove residual reagent and dry the columns, they were centrifuged again for 1 min at 13,000 ×***g*** and the catch tube discarded and replaced with a clean microtube. To remove the RNA bound to the column, 50 µl of RNase-free water was added directly to the centre of each column and incubated at room temperature for 5 min before being centrifuged at 13,000 ×***g*** for 1 min. The flow-through was placed on ice and the column transferred to a clean microtube. A fresh 50 µl of RNase-free water was added to the column and incubated at room temperature for 5 min before the elution was repeated. The RNA quantity and purity in the elutions were assessed using a Nanodrop ND1000 spectrophotometer. First and second elutions of acceptable purity and quantity from each sample were combined and treated with an in-solution DNase digest step using combined 80 µl of elution, 10 µl of DNase and 10 µl of buffer RDD from the Qiagen RNeasy PowerBiofilm kit. This was incubated at room temperature for 20 min before being re-bound to the spin column using the PB solution and ethanol and subsequently washed with WB and PB solutions as described above. The final purified RNA was eluted in 50 µl of RNase-free water. The quantity and purity of the elution was tested via Nanodrop.

### RNA sequencing

RNA sequencing was carried out on the Illumina platform at Deepseq (University of Nottingham, UK). Dry ice was used for shipment of the RNA samples to Deepseq, where the sequencing facility reported that the samples were quality-checked again using an Agilent TapeStation 4200 and the Agilent RNA ScreenTape Assay Kit (Agilent, USA). Following this, a Qubit RNA BR assay kit from Life Technologies was used to quantify the total concentration of the RNA samples so that an aliquot of 1 µg of total RNA could be used for rRNA depletion using the QIAseq FastSelect –5S/16S/23S kit (Quiagen, Germany). A NEBNext Ultra II Directional RNA library prep kit for Illumina (NEB, USA) was then used with NEBNext^®^ Multiplex Oligos for Illumina^®^ (96 Unique Dual Index Primer Pairs) to create the whole transcriptome indexed libraries. After second-strand synthesis using eight cycles of PCR, the libraries were quantified using a Qubit Fluorometer with a Qubit dsDNA HS Kit (Thermo Fisher Scientific, USA). Library fragment-length distributions were analysed using the Agilent TapeStation 4200 and the Agilent High Sensitivity D1000 ScreenTape Assay (Agilent). Libraries were pooled in equimolar amounts and quantified using the KAPA Library Quantification kit for Illumina (Roche, Germany). The pooled library was then loaded onto the Illumina NextSeq 500, on a NextSeq 500 Mid Output v2.5 150 cycle kit (Illumina, USA), to generate ~10 million 75 bp paired-end reads per sample. RNA sequencing was performed on three biological replicates for each strain in biofilm and planktonic mode of growth, allowing DESeq to estimate gene-wise variability across replicates and improve modelling of expression counts for differential expression analysis.

### Differential gene expression analysis

Raw sequencing reads were trimmed to remove Illumina adapters and low-quality nts (Q<20) using Trim Galore (v0.6.7), and reads shorter than 20 bp were discarded. Trimmed reads were aligned to the appropriate *Clostridium difficile* reference genome using HISAT2 (v2.2.0). StringTie (v2.2.1) was used for transcript assembly and gene-level quantification. Raw gene-level read counts were supplied to DESeq2 (v1.34.0) for differential expression analysis. DESeq2 normalization was performed internally by estimating sample-specific size factors using the median-ratio method, followed by dispersion estimation and statistical testing using the default settings. Resulting *P* values were corrected for multiple testing using the Benjamini–Hochberg method, and genes with an adjusted *P* value<0.05 were considered differentially expressed. No minimum log_₂_ fold-change (log_2_FC) threshold was applied. Seven DESeq2 gene expression contrasts were performed to identify the differences in gene expression between each strain grown as a colony biofilm compared to planktonic growth and between mutant and parent strains. Data generated from RNA sequencing including HTSeq counts are available in the NCBI GEO repository, accession no. GSE336654. Full gene lists with log_2_FCs per contrast are available as supplementary data (Table S3).

### Gene set enrichment analysis by KEGG and biological processes

Gene set enrichment analysis (GSEA) was used to identify KEGG pathways and biological processes (BPs) from Gene Ontology (GO) that were significantly enriched in each dataset. KEGG pathway gene sets for *C. difficile* 630 (organism code: cdf) and *C. difficile* 630Δ*erm* (organism code: pdf) were retrieved in R using KEGGREST, and the associated genes were matched to those present in the expression datasets for each strain. GO annotations were downloaded from QuickGO for *C. difficile* 630 (Taxon: 272563) and mapped to *C. difficile* 630Δ*erm* using the old locus-tag gene identifiers shared between the two strains. Genes were ranked by log_2_FC prior to enrichment analysis. No minimum log_2_FC threshold was applied.

### Quantitative Real-Time PCR (qPCR) validation

Primers used in this study were selected for genes involving key virulence factors in *C. difficile,* including motility, toxin production and sporulation, with reference genes *adk, rpsJ* and *gyrA* (Table S2). The primer efficiencies were tested using cleaned-up PCR products generated from each primer pair. A fivefold dilution series was created from PCR products in nuclease-free water. A master mix of Sybr Green Master Mix 10× (Roche, Germany) and nuclease-free water was aliquoted into microfuge tubes for each primer pair being tested. The primers were added from a working stock containing 10 mM forward and 10 mM reverse primers to a final concentration of 1 mM. The master mix for each primer set was aliquoted into PCR tubes, one for each dilution being tested and one for each of the no-template controls containing nuclease-free water. To each tube, the template or nuclease-free water control was added in aliquots of 3 µl before being vortexed briefly to mix and centrifuged gently for 30 s at 5,000 ×***g*** to gather all liquid to the bottoms of the tubes. The mixture was aliquoted into three technical replicates per dilution onto the qPCR plate.

qPCR was carried out on a Lightcycler^®^ 480 (Roche, Germany) with an initial denaturation step of 95 °C for 5 min, followed by 50 cycles of denaturation at 95 °C for 10 s, primer annealing at 57 °C for 10 s and then an extension step of 72 °C for 30 s. A melting curve was performed with all runs from 65 °C to 97 °C to assess the quality of each run.

To calculate the percentage primer efficiency, the technical replicates for each dilution were averaged and the log value of sample quantity calculated from the dilution. The slope of log sample quantity against average threshold cycle (Ct) value was calculated using the formula below in Microsoft Excel.

=SLOPE(Average Ct value range, log quantity range)

The percentage primer efficiency was then derived from the calculated ‘slope’ using the following formula:

(10^(−1/Slope)^−1)×100

Reverse transcription of extracted RNA was performed by using 500 ng of RNA and 50 ng of random primers (Promega, UK) made up to 12 µl using nuclease-free water. This was incubated for 10 min at 70 °C. After incubation, 4 µl of 5× first strand buffer, 2 µl of 0.1 M DTT mix and 1 µl of 10 mM dNTPs mix containing (dATP, dTTP, dCTP and dGTP) to a final concentration of 0.2 mM were added with 1 µl of RNaseOUT (Invitrogen, USA). A further incubation for 2 min at 42 °C was performed before 200 U of reverse transcriptase was added and incubated first for 50 min at 42 °C, then a further 15 min at 70 °C. All reactions were performed alongside negative controls for each RNA sample, which contained no reverse transcriptase enzyme. End-point PCR was performed on all reverse transcription reactions and controls with *adk* reference gene primers (Table S1) and visualized using gel electrophoresis to validate that the reverse transcription reaction was efficient.

qPCR was performed on all RNA samples diluted 1 : 10 in RNase-free water using the same cycle conditions and master mix as previously described. A separate qPCR plate was used for each of the validated qPCR primers.

Standard curves were generated for each primer set using the same qPCR cycle conditions as described above. For each primer set, a pooled cDNA sample from the experimental condition with the lowest average Ct values was selected. This ensured that the lowest possible Ct values were included in the standard curves, and the dynamic range of Ct values included was wide enough to cover all samples. The pooled cDNA was first diluted 1 : 10 before being diluted fivefold and added to the qPCR master mix as previously described.

The percentage primer efficiencies were calculated as previously described before being converted to a different scale using the formula below for calculation of relative gene expression:

Converted primer efficiency=[primer efficiency (%)/100]+1

The Pfaffle method [19] was used to calculate relative gene expression for each sample with multiple housekeeping genes, using the formula below, where *E* is the converted primer efficiency, GOI refers to ‘gene of interest’ and REF refers to ‘reference gene’:


$$Relative\;Gene\;Expression=\frac{(E_{GOI}{)}^{\Delta Ct\;GOI}}{GeoMean[(E_{REF}{)}^{\Delta Ct\;REF}]}$$

## Results

### *C. difficile* colony biofilms

Visible differences in colony biofilm structure could be observed by eye for the 630Δ*erm:dnaK* strain, which appeared shrivelled and had reduced extracellular polymeric substance compared to the parent and wild-type strains. To assess a hypothesized structural difference in more detail, colony biofilms of strain 630, 630Δ*erm* and 630Δ*erm:dnaK* were grown on mixed nitrocellulose ester membranes for 24 h prior to being imaged by low vacuum scanning electron microscopy (SEM) with an LFD detector. The structure of the 630 and 630Δ*erm* 24 h colony biofilms was similar at all magnifications (Fig. 1). Many tears in the relatively smooth surface of the biofilm revealed a complex biofilm matrix within. At a higher magnification (12,000×), the cells on the surface of the 630 and 630Δ*erm* colony biofilms appeared very tightly packed with minimal gaps between them. However, the structure of the 630Δ*erm:dnaK* colony biofilm did not exhibit the same pattern of tearing on the biofilm surface. With the mutant, the tears were much longer and apparently connected but were relatively thin in comparison to the ‘tears’ of the parent and wild-type biofilms. At 12,000× magnification, the 630Δ*erm:dnaK* cells were faintly visible as an apparently more irregular network under the surface of the biofilm, with some small gaps forming between the cells. Overall, the *dnaK* mutant biofilms appeared denser, with the lengthened cells tangled and shrouded in a thicker matrix.

**Fig. 1.**
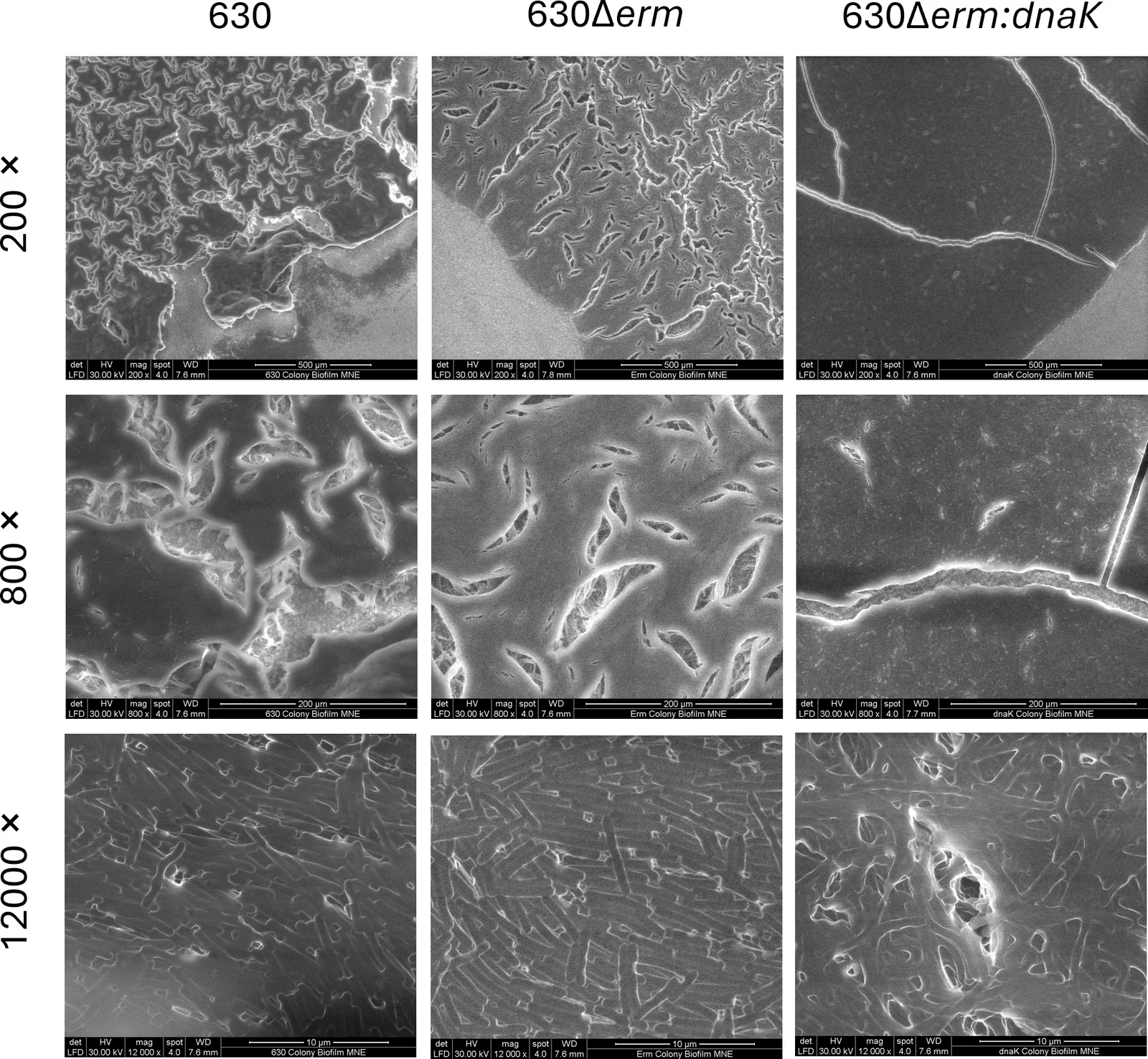
*C. difficile* 630, 630Δ*erm* and 630Δ*erm:dnaK* 24 h colony biofilms cultured on semi-permeable 13 mm ᴓ mixed nitrocellulose ester membranes with pore size 0.2 µm and imaged by low-vacuum SEM equipped with an LFD at 200×, 800× and 12,000× magnification.

### Overall differential gene expression per contrast

Seven differential gene expression comparisons were performed between mutant and parent strain under the same growth conditions and between growth conditions within the same strains. RNA sequencing data were confirmed by qPCR validation (Table S3). *C. difficile* 630Δ*erm* had only 6.5% of genes statistically significantly differentially expressed under planktonic conditions compared to the wild-type 630 (Fig. 2). Whereas when grown in a biofilm, the same strains differed by 40.8% of the transcriptome. Disruption of the *dnaK* gene in 630Δ*erm:dnaK* resulted in a 52.8% change in the transcriptome compared to 630Δ*erm* under planktonic growth, whereas this increased to a 64.0% difference when the strains were grown as colony biofilms.

**Fig. 2.**
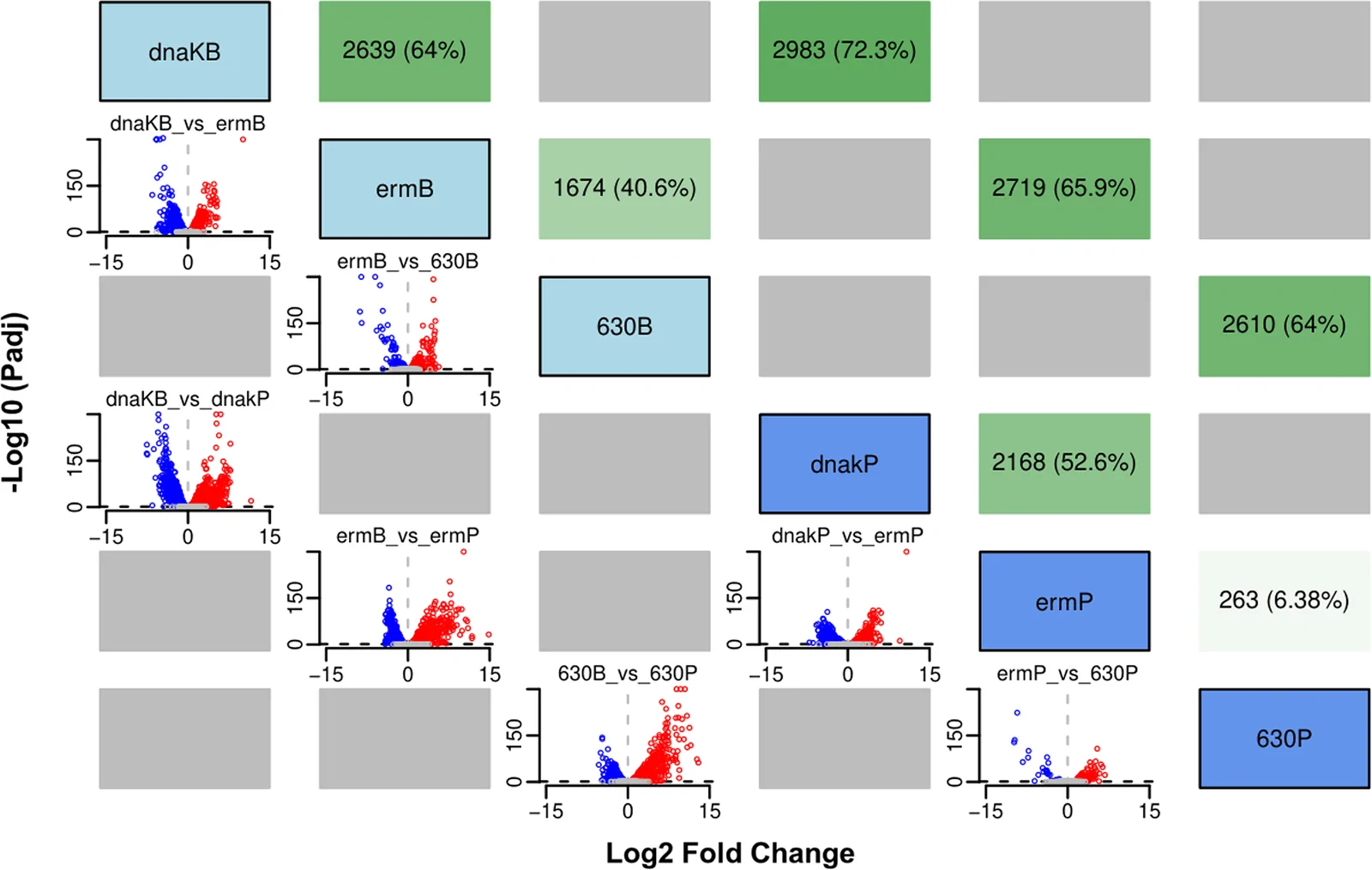
Differential gene expression between *C. difficile* strains under planktonic and biofilm growth conditions. Differential expression was compared between strains 630 (630), 630Δ*erm* (erm), and 630Δ*erm:dnaK* (dnak), cultured planktonically (p) or as 24 h colony biofilms (b). Diagonal boxes identify the strain and growth condition included in each comparison. Lower-left panels show volcano plots of DESeq2-estimated log_2_FC (horizontal-axis) against −log_10_ Benjamini–Hochberg-adjusted *P* value (vertical-axis). For contrasts labelled A_vs_B, positive log_2_FC values indicate higher expression in A relative to B, whereas negative values indicate lower expression in A relative to B. Genes with an adjusted *P* value<0.05 are shown in red when upregulated in A and blue when downregulated in A; genes not meeting this criterion are shown in grey. No minimum log_2_FC threshold was applied. Upper-right panels report the number and percentage of genes with an adjusted *P* value<0.05 in each comparison. Percentages were calculated relative to the total number of genes tested. Grey panels indicate comparisons that were not performed.

When comparing biofilm to planktonic growth for each of the strains, 630 and 630Δ*erm* showed similar changes in transcriptome with 63.2 and 66.0%, respectively. 630Δ*erm:dnaK,* when grown in a colony biofilm compared to planktonic growth showed the biggest change in transcriptome with 72.4% of genes statistically significantly differentially expressed (*P*_adj_<0.05).

Across the three strains, the biofilm-to-planktonic contrasts revealed a strong shared transcriptional response, with a core set of 634 genes consistently significantly increasing in expression and 625 consistently significantly decreasing in expression (Fig. 3a). However, the 630Δ*erm:dnaK* biofilm-to-planktonic contrast has a large number of differentially expressed genes falling outside this shared core. There were 301 genes that were uniquely significantly increased when compared to the other biofilm-to-planktonic contrasts and 260 genes that were uniquely significantly decreased. Genes belonging to each group can be identified in the gene-membership tables (Tables S4–6). Also notably, 183 genes are significantly decreased in a 630Δ*erm:dnaK* biofilm contrast, where they are increased in 630 and 630Δ*erm*, and oppositely, 153 are increased in a 630Δ*erm:dnaK* biofilm when decreased in 630 and 630Δ*erm*.

**Fig. 3.**
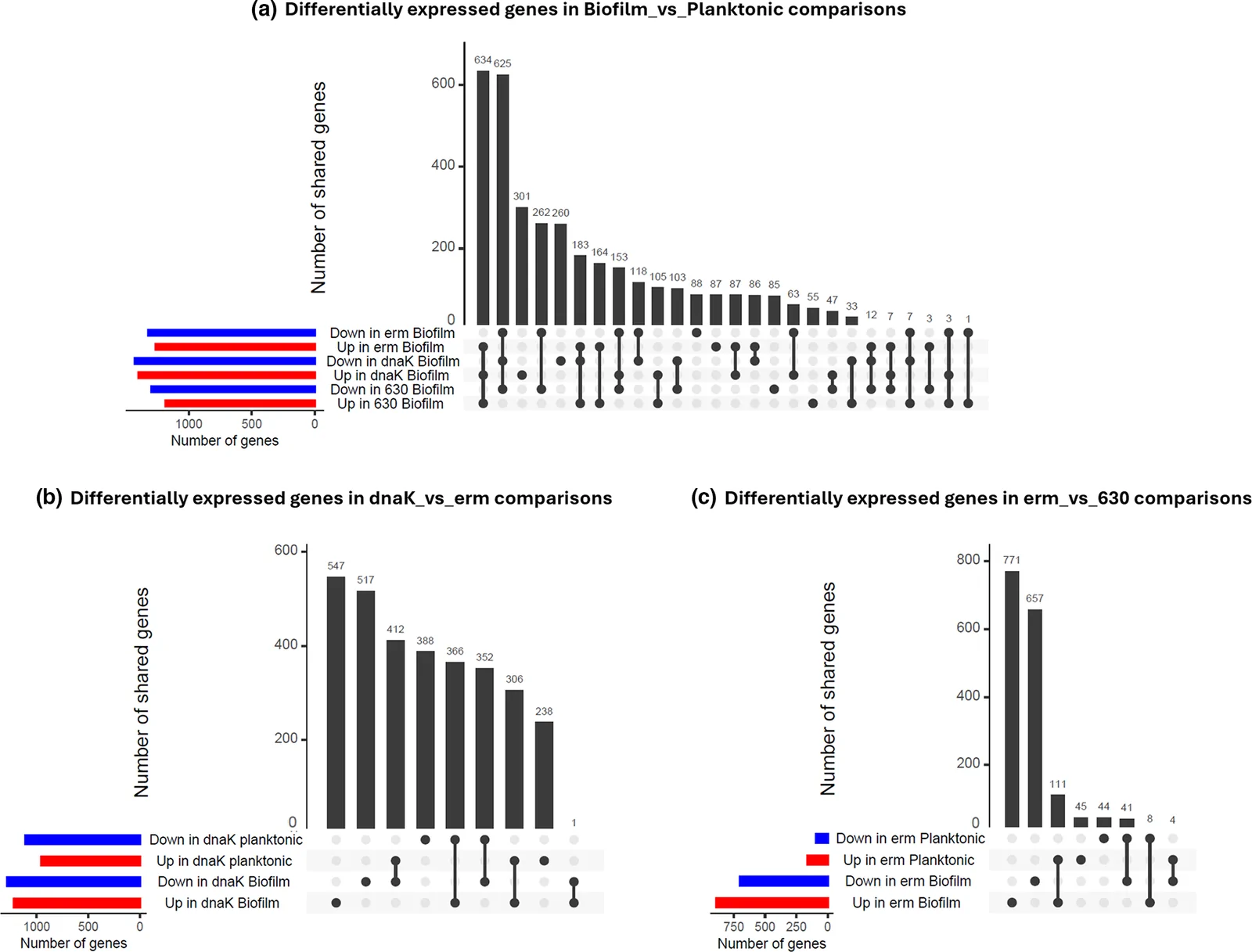
Overlap of significantly differentially expressed genes between *C. difficile* strains under planktonic and biofilm growth conditions. UpSet plots showing the overlap and uniqueness of significantly upregulated and downregulated genes across three sets of comparisons: (**a**) biofilm versus planktonic growth in the 630, 630Δ*erm* and 630Δ*erm:dnaK* strains; (**b**) 630Δ*erm:dnaK* versus 630Δ*erm* under biofilm and planktonic conditions; and (**c**) 630Δ*erm* versus 630 under biofilm and planktonic conditions.

When comparing 630Δ*erm:dnaK* to 630Δ*erm* under either planktonic or biofilm conditions, a large portion of genes were significantly differentially expressed only in a biofilm (547 increased and 517 decreased), and a large number (352 decreased and 306 increased) were significantly differentially expressed regardless of the conditions (Fig. 3b). When considering the 630Δ*erm* compared to 630, relatively few genes were significantly differentially expressed in planktonic conditions; however, a large number were differentially expressed (771 increased and 657 decreased) when forming a biofilm (Fig. 3c).

### GSEA

GSEA of all contrasts were compared not only to identify patterns of changing pathways when forming a biofilm but also to identify differences in gene expression between strains under the same conditions. GSEA identifies clusters of coordinated gene expression where genes belonging to a specific pathway are significantly concentrated in a positive or negative direction without applying a differential expression significance cutoff. As a baseline, comparing gene expression in *C. difficile* 630 colony biofilms to planktonically cultured cells revealed 15 KEGG pathways and 7 BPs that were enriched in this contrast (Fig. 4). The only category that was significantly increased in expression was ‘sporulation resulting in formation of a cellular spore’. The categories that were decreased in expression centred around metabolic processes. When gene expression in *C. difficile* 630Δ*erm* colony biofilm was compared to planktonically cultured cells, a very similar pattern emerged with focus on the same categories combined with the addition of a decrease in bacterial-type flagellum-dependent cell motility and bacterial-type flagellum organization, as well as decreases in some additional metabolic processes including selenocompound metabolism, pyrimidine metabolism and porphyrin metabolism (Fig. 4). In contrast, when comparing gene expression in *C. difficile* 630Δ*erm:dnaK* colony biofilms to planktonically cultured cells, there were double the number of KEGG pathways (30 in total) that were enriched, with over half of them being unique to *C. difficile* 630Δ*erm:dnaK* biofilm formation when compared to the parent and wild-type strains. There were nine core pathways enriched in all strains forming a colony biofilm and were involved with metabolic processes and flagellar assembly. The additional KEGG pathways that are enriched in 630Δ*erm:dnaK* are heavily involved with carbon fixation, amino acid biosynthesis and remodelling of the cell wall. Peptidoglycan synthesis was increased in expression in 630Δ*erm:dnaK* colony biofilms, where no other KEGG pathways were positively enriched in the parent or wild-type strains forming a biofilm.

**Fig. 4.**
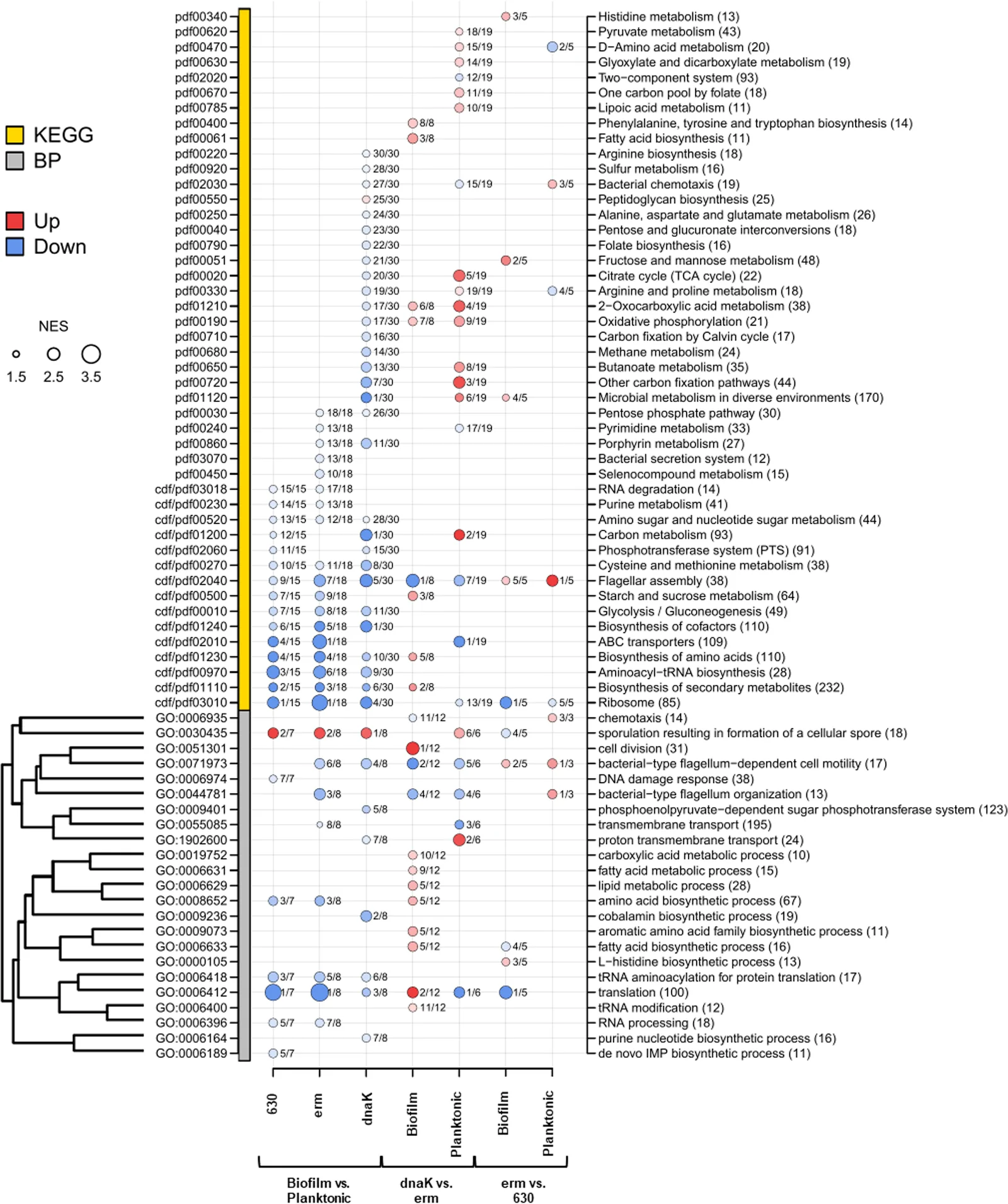
GSEA of *C. difficile* strains under planktonic and biofilm growth conditions. GSEA was performed on transcriptomic comparisons of *C. difficile* strains 630, 630Δ*erm* (erm) and 630Δ*erm:dnaK* (dnaK) cultured planktonically or as a 24 h colony biofilm to identify enriched KEGG pathways and GO BPs terms associated with growth condition and strain differences. Fractions notated beside each data point represent the rank of KEGG pathway or GO by NES (normalized enrichment score), showing from 1 /*N*, most enriched pathway to *N*/*N*. *N*=number of pathways enriched.

When comparing 630Δ*erm:dnaK* colony biofilms to parent strain 630Δ*erm* colony biofilms, there were eight KEGG pathways, which were all positively enriched except for flagellar assembly, which was negatively enriched. The eight pathways that were positively enriched included biosynthesis of amino acids, specifically phenylalanine, tyrosine and tryptophan, as well as fatty acids and secondary metabolites, alongside oxidative phosphorylation and both 2-oxocarboxylic acid and starch and sucrose metabolism. There were also 12 BPs enriched, which were mostly positively enriched, with 3 being negatively enriched. The three negatively enriched BPs were all involved with motility, namely chemotaxis, bacterial-type flagellum-dependent cell motility and bacterial-type flagellum organization. The largest enriched BP in this comparison was cell division, followed by translation, then equally multiple metabolic processes including lipid metabolic process, amino acid biosynthetic process, fatty acid biosynthetic process and aromatic amino acid family biosynthetic process. The differences in transcription profiles of 630Δ*erm:dnaK* colony biofilms compared to parent strain 630Δ*erm* colony biofilms were not reflected when the same strains were compared in planktonic growth. More metabolic pathways were differentially expressed in the planktonically grown culture comparison, with only three KEGG pathways showing similar enrichment patterns to the biofilm comparison, being a negative enrichment in flagellar assembly and a positive enrichment in both 2-oxocarboxylic acid metabolism and oxidative phosphorylation. Carbon metabolism was positively enriched, when planktonic 630Δ*erm:dnaK* were compared to 630Δ*erm* under the same conditions. However, this pathway is not enriched in 630Δ*erm* forming a biofilm and is strongly decreased in expression in 630Δ*erm:dnaK* forming a biofilm. This same pattern was reflected in five other KEGG pathways, namely, TCA cycle, arginine and proline metabolism, butanoate metabolism, other carbon fixation pathways and microbial metabolism in diverse environments. Five KEGG pathways were enriched exclusively in the 630Δ*erm:dnaK* to 630Δ*erm* planktonic comparison, one of which, the two-component system, was decreased in expression, whereas lipoic acid metabolism, one-carbon pool by folate, glyoxylate and dicarboxylate metabolism and pyruvate metabolism were all increased in expression.

The remaining two comparisons, between 630 and 630Δ*erm* when grown in a biofilm and when grown planktonically, had the least number of enriched categories. When grown in a biofilm, five KEGG pathways and five BPs were enriched in the comparison between 630 and 630Δ*erm*. The five KEGG pathways included positive enrichment of histidine metabolism, fructose and mannose metabolism, microbial metabolism in diverse environments and flagellar assembly with only ribosome KEGG pathway negatively enriched. The five BPs reflected this with a positive enrichment of l-histidine biosynthesis and bacterial type flagellar dependent cell motility, with a negative enrichment in sporulation, fatty acid biosynthesis and translation. When grown planktonically, five KEGG pathways and three BPs were enriched in the comparison between 630 and 630Δ*erm*. Notably, the three BPs that were enriched in 630Δ*erm* planktonic cells, when compared to 630, were all motility-related and all positively enriched. This is also reflected in the increased expression of the flagellar assembly and bacterial chemotaxis KEGG pathways in 630Δ*erm* planktonic cells when compared to 630.

## Discussion

*C. difficile* biofilms are increasingly recognized as spore reservoirs contributing to persistence and recurrence of CDI [1, 2, 7]. Understanding the regulatory networks that promote biofilm development may help to identify pathways that could be targeted to reduce this disease persistence. The phenotypic effects of disruption to the *dnaK* gene in *C. difficile* have been extensively reported to demonstrate a link between the stress response and biofilm formation in *C. difficile* [17, 20, 21]. The transcriptomic analysis in this paper reveals the specific pathways that are affected by disruption of the *dnaK* gene and closely align with known pathways of biofilm formation.

Initial colony biofilm investigations found that disruption of the *dnaK* gene resulted in structural differences in colony biofilm morphology at 24 h (Fig. 1). The 24 h 630Δ*erm:dnaK* colony biofilm structure had longer and apparently connected tears in the surface, which were relatively thin in comparison to the parent and wild-type colony biofilms at 24 h. Similar structural differences were previously observed when *C. difficile* 630, 630Δ*erm* and 630Δ*erm:dnaK* were grown as aggregate biofilms on glass coverslips and imaged by high-vacuum SEM [20]. These findings reveal that disruption of the *dnaK* gene in *C. difficile* 630Δ*erm* causes similar biofilm phenotypic traits regardless of the method of biofilm culture.

Transcriptomic analysis showed that disruption of the *dnaK* gene caused widespread metabolic and transcriptional reprogramming in comparison to the parent strain 630Δ*erm*. A coordinated upregulation of metabolic processes of the *dnaK* mutant in planktonic culture was seen increased expression in 13 KEGG pathways, including metabolism of pyruvate, d-amino acids, lipoic acids, butanoate and carbon. This higher rate of metabolic processes in 630Δ*erm:dnaK* aligns with increased metabolism due to a dysregulated stress response and/or a delay in achieving stationary phase [17, 22]. Molecular chaperone proteins DnaJK are both transcriptionally and proteomically increased in expression in *C. difficile* 630Δ*erm* when in stationary phase [22]. Therefore, we propose that 630Δ*erm:dnaK* is delayed in metabolically achieving stationary phase due to the lack of efficient chaperone proteins. Alternatively, it is physiologically possible that the cells are both delayed in growth phase alongside experiencing a stress response. This is supported by the reduced planktonic growth of 630Δ*erm:dnaK* at 37 °C previously reported, which was originally hypothesized to be a result of a dysregulated heat stress response in the cells [17].

After 6 h of planktonic growth, cultured 630Δ*erm:dnaK* cells expressed increased oxidative phosphorylation as well as sporulation as a BP, in contrast to 630Δ*erm* under the same conditions. Spore formation is a known stress response as a way of survival and is also strongly associated with biofilm formation [8, 9]. Sporulation is an energy intensive process where phosphorylation of key regulatory factors, such as Spo0A, plays a critical role in initiation [10, 23]. This premature increased expression of genes associated with the sporulation pathway in planktonic 630Δ*erm:dnaK* suggests earlier initiation of spore formation, which may result from the heightened stress response in this strain, leading to earlier biofilm initiation. Of note, functional spores have not been recoverable from the DnaK-deficient strain [20, 24]. Therefore, although the genetic pathway for sporulation has been increased in expression, physical spores have not been observed, indicating a problem with production of the spore proteins or spore assembly. These cells are either failing to sporulate post-transcriptionally or, as is seen in *Bacillus subtilis*, the alternative phosphorylation of Spo0A could have redirected cellular pathways towards early biofilm formation [23].

Of particular interest in the transcriptome of 630Δ*erm:dnaK* colony biofilm cells, compared to 630Δ*erm*, was a significantly increased enrichment of cell division, which was not observed in any other contrast. This, combined with the increased translation, tRNA modification and other biosynthetic and metabolic processes in the 630Δ*erm:dnaK* biofilm cells, could indicate that the *dnaK* mutation has resulted in a cellular attempt to increase growth and proliferation in a biofilm compared to 630Δ*erm* under the same conditions. Rapidly proliferating cells in the 630Δ*erm:dnaK* biofilms could have explained the increased biofilm; however, previous studies found no significant increase in cell number in 630Δ*erm:dnaK* aggregate biofilms compared to the parent strain [20]. Therefore, the question remains whether the transcriptionally increased cell division pathways are phenotypically successful in the *dnaK* mutant. A post-transcriptionally failed cell division process could be attributed to the 50% longer cell phenotype observed in 630Δ*erm:dnaK* in comparison to the parent and wild-type strain. However, since this phenotype is also observed in planktonic 630Δ*erm:dnaK* cells [17], it would have been expected to see an increase in cell division in the 630Δ*erm:dnaK* to 630Δ*erm* planktonic contrast.

A strong coordinated reduction in motility genes in 630Δ*erm:dnak* biofilm cells compared to 630Δ*erm* was noted by significant enrichment in flagellar assembly, chemotaxis, bacterial-type flagellum-dependent cell motility and bacterial-type flagellum organization. This lack of motility aligns with phenotypical observations of the *dnaK* mutant [17, 20] as well as enhanced biofilm formation, as it is well established that genes associated with flagella are decreased in *C. difficile* biofilm formation [25, 26]. In *C. difficile* mutant cells where either flagellar assembly or post-translational modifications have been altered, biofilm formation has been affected [13, 27]. For example, multiple different modifications in flagellar assembly of *C. difficile* R20291 led to increased cell hydrophobicity, which was concluded to be responsible for increased biofilm formation via increased aggregation [27]. Similarly, a restructuring of the cell surface suggested to cause increased hydrophobicity in 630Δ*erm:dnaK* cells [21], aligns with significantly increased enrichment in peptidoglycan synthesis in 630Δ*erm:dnaK* biofilms compared to planktonic cells.

The 630Δ*erm* planktonic cells also showed a significant increase in five pathways relating to motility and chemotaxis compared to 630, which has not been phenotypically observed in previous work [28, 29]. Conversely, four out of five of these motility-related pathways were significantly decreased in DnaK-deficient planktonic cells compared to 630Δ*erm*. Taken together, these observations strongly suggest that disruption of the *dnaK* gene led to a transcriptional shift in motility towards a sessile cellular state seen in biofilm initiation.

Overall, loss of erythromycin resistance in 630Δ*erm* when cultured planktonically resulted in five KEGG pathways and three BPs that were significantly enriched compared to the wild-type 630. Previous genomic comparison of 630Δ*erm* and wild-type 630 found multiple genomic variations in addition to the loss of erythromycin resistance [29]. None of these variations were directly associated with motility or chemotaxis and therefore cannot easily explain the transcriptomic changes identified in these pathways in this work [29]. However, due to the high quantity of mobile genetic elements in *C. difficile* 630 [30], it is entirely possible that subculturing of *C. difficile* 630Δ*erm* by different labs may have led to variations in different lab strains. Importantly, the 630Δ*erm* strain used in this study was the parent strain used to derive the 630Δ*erm:dnaK.* Therefore, we can determine that the transcriptional differences in 630Δ*erm:dnaK* should not be due to other genomic differences in 630Δ*erm* [17].

## Conclusion

This work generated the first global transcriptional description of a DnaK-deficient *C. difficile* strain grown as a colony biofilm and identified several pathways linking the stress response to increased biofilm formation. A core number of KEGG pathways did remain enriched in all strains, including a decrease in flagellar assembly, as well as decreases in biosynthesis of amino acids, aminoacyl-tRNA, secondary metabolites, cofactors and ribosomes and a reduction in glycolysis/gluconeogenesis. This is reflected in the BPs, where tRNA aminoacylation for protein translation and translation were reduced in all strains forming a biofilm. This paints a very clear picture that reduction in protein metabolism in biofilms is a core process as cells enter a sessile state. Perhaps by disruption of the *dnaK* gene, producing a key molecular chaperone for protein folding, this has promoted an active stress response together with reduced translation, leading to earlier and enhanced biofilm formation [17, 20]; via eradication of motility processes, forcing cells into a sessile state alongside premature upregulation of sporulation-associated genes at 6 h planktonic growth. Further proteomic analysis would generate a fuller picture of how disruption of the *dnaK* gene affects translation and protein folding. However, these findings support a role for *dnaK* in coordinating stress adaptation and biofilm-associated physiology and provide a framework for future mechanistic studies investigating persistence-related phenotypes in *C. difficile*.

## Supplementary material

10.1099/jmm.0.002212 Supplementary Material 1.

10.1099/jmm.0.002212 Supplementary Material 2.

## Abbreviations

BHI: brain heart infusion
BHIS: brain heart infusion-supplemented
BP: biological process
CDI: *C. difficile* infection
ESEM: environmental scanning electron microscope
GEO: Gene Expression Omnibus
GO: Gene Ontology
GSEA: gene set enrichment analysis
KEGG: Kyoto Encyclopedia of Genes and Genomes
LFD: large field detector
log_2_FC: log_₂_ fold-change
NCBI: National Center for Biotechnology Information
SEM: scanning electron microscopy
