# Supplementary material for: Disruption of dnaK results in transcriptional reprogramming of biofilm-associated pathways in Clostridioides difficile
Source: J Med Microbiol. 2026 Oct 7;75(10):002212. doi: 10.1099/jmm.0.002212 (PMC13645377; doi:10.1099/jmm.0.002212)
Supplement: Supplementary file 1 [file jmm-75-02212-s001.pdf]

Supplementary Material

Supplementary table 2. Primers used for qPCR

Gene	Primers	Primer sequences 5'-3'	Product size (bp)	T_a (ºC)	Reference
adk	adk-F	GTGTATGTGATGTATGCCAAG	196	57	Metcalf *et al.*, 2010
	adk-R	CCTAAGGCTGCGACAATATC			
gyrA	gyra-F	CTCGTATTGTTGGGGACGTT	146	57	Deneve *et al.*, 2008
	gyra-R	ATCCCCATCAACAGAACCAA			
rpsJ	rpsj-F	GATCACAAGTTTCAGGACCTG	151	57	Metcalf *et al.*, 2010
	rpsj-R	GTCTTAGGTGTTGGATTAGC			
fliC	flic-F	TGATGATGCTGCTGGACTTG	119	57	Moore, 2020
	flic-R	ACGAACCTTCTGCTGTTTGTAC			
fliD	flid-F	AGCTGGACAAATTGCCAGTG	114	57	Moore, 2020
	flid-R	CCTTGGTCATCAGTTACATCAGC			
tcdA	tcda-F	AGCTTTCGCTTTAGGCAGTG	129	57	Moore, 2020
	tcda-R	ATGGCTGGGTTAAGGTGTTG			
spoVB	spovb-F	ATTCAGGGAATGGGAAAACC	161	57	Moore, 2020
	spovb-R	TTAATCATGGCTGCCACAAA			
spoIIIAC	spoiiiac-F	ATCATTGATATTAAAGGTTGCAGGT	150	57	Moore, 2020
	spoiiiac-R	TCACTTATTTCTGTTATTACCATACCC			
cspC	cspc-F	AGTGGTGCAGGAAATCAAGG	155	57	Moore, 2020
	cspc-R	CTGTGCTCCCACTTTATCTGG			

Supplementary table 3. Comparison of Log_2FC in gene expression as measured by qPCR and by RNA sequencing.

Contrast	Method	Log_2FC						r	p-value
		fliC	*fliD*	*tcdA*	*spoIIIAC*	*spoVB*	*cspC*		
630 biofilm vs planktonic	qPCR	-0.9	-1.9	-0.1	10.5	1.1	6.7	0.99	0.000
	RNAseq	-2.9	-2.8	-1.4	7.5	<u>0.3</u>	5.5		
630Δ*erm* biofilm vs planktonic	qPCR	-1.9	-2.2	3.1	11.2	0.9	6.1	0.98	0.001
	RNAseq	-3.8	-3.5	1.5	7.4	<u>-0.1</u>	5.3		
630Δ*erm:dnaK* biofilm vs planktonic	qPCR	-1.6	-2.1	-1.6	8.1	1.0	5.0	0.97	0.002
	RNAseq	-1.3	-1.6	-1.3	5.6	1.5	5.7		
630Δ*erm* vs *630* biofilms	qPCR	2.4	2.2	5.3	0.1	0.5	-0.2	0.99	0.000
	RNAseq	2.0	1.8	4.7	<u>0.1</u>	-0.4	-0.3		
630Δ*erm:dnaK* vs 630Δ*erm* biofilms	qPCR	-2.8	-3.2	-6.7	-1.4	0.1	-0.2	0.99	0.000
	RNAseq	-1.4	-1.7	-5.2	-0.9	1.3	0.8		
630Δ*erm* vs 630 planktonic	qPCR	3.4	2.5	2.1	-0.2	0.4	0.4	0.98	0.001
	RNAseq	3.0	2.5	1.8	<u>0.1</u>	<u>0.0</u>	<u>0.1</u>		
630Δ*erm:dnaK* vs 630Δ*erm* Planktonic	qPCR	-3.2	-3.3	-2.0	1.8	0.1	-0.2	0.99	0.000
	RNAseq	-3.9	-3.7	-2.4	<u>0.8</u>	<u>-0.4</u>	<u>-0.3</u>		

Blue text indicates absolute fold changes which decreased in gene expression between conditions tested. Red text indicates absolute fold changes which increased in expression. Data underlined are not significantly different as determined by RNA sequencing *padj* >0.05. Association between datasets was assessed via Pearson correlation to show strength and direction of the relationship by r values and associated p-values.

References

Denève C, Deloménie C, Barc M-C, Collignon A, Janoir C. Antibiotics involved in *Clostridium difficile*-associated disease increase colonization factor gene expression. *J Med Microbiol* 2008;57:732–738. 10.1099/jmm.0.47676-0

Metcalf D, Weese JS. Evaluation of commercial kits for extraction of DNA and RNA from *Clostridium difficile*. *Anaerobe* 2012;18:608–613. 10.1016/j.anaerobe.2012.10.003

Moore N. *Transcriptomic Analysis of Clostridioides Difficile Grown in a Biologically Relevant Growth Medium*. Ulster University, 2020.
